# A case control study investigating factors associated with high infant death in Saiha district of Mizoram, India bordering Myanmar

**DOI:** 10.1186/s12887-017-0778-z

**Published:** 2017-01-17

**Authors:** Alok K. Deb, Shanta Dutta, Chhaihlo Hnichho, Mary Vanlalpeki, Hli Thapi Phosa, Khaila Rakhu, Samuel Lalfakawma Fanai, Manoj Chakrabarti, Samiran Panda

**Affiliations:** 1 National Institute of Cholera & Enteric Diseases / Indian Council of Medical Research (NICED/ICMR), P-33 CIT Road, Scheme-XM, Beliaghata, Kolkata, 700010 India; 2 District Health Program Administration, Saiha, Mizoram India; 3 National Health Mission, Saiha, Mizoram India; 4 District Hospital, Saiha, Mizoram India

**Keywords:** Infant mortality, Mizoram, Areca nut, Low birth weight, Child delivery at home

## Abstract

**Background:**

Infant mortality has dropped considerably in India over the last 5 years. A sharp contrast to this decline in national average of infant mortality is the rate recorded during 2014–2015 from the southernmost district of Saiha, Mizoram having a common international border with Myanmar. As this district specific rate (113 per 1000 live births) is 3 times higher compared to the national and state average, the present investigation was carried out to identify associated factors.

**Methods:**

We examined secondary data made available by the national health mission, consulted with local community members and generated primary data through interviews. A case-control study design was followed. Mothers, who delivered a child during 2013–2015 and subsequently lost them due to infant death, formed the case group and controls were selected from same neighborhood as with case-mothers. The mother and child tracking system maintained by the district specific national health mission office was used for recruiting cases and controls. A total of 195 mothers were interviewed; 66 of them belonged to ‘cases’ and 129 were ‘controls’.

**Results:**

The mean age of the respondents was 27 years (median 27; SD ± 5; minimum 17 & maximum 44). In uni-variate analyses ‘child delivery at home’, ‘low birth weight’, ‘non-attendance of school by mothers’, ‘completed standard of school education by mothers’, ‘both parents working’, ‘mothers receiving blood transfusion during last pregnancy’, and ‘fourth or more birth order during last pregnancy’ were associated with infant deaths. Intriguingly, the number of daily *kuhva* (raw areca nut) intake during last pregnancy was significantly higher among case-mothers compared to controls. In conditional logistic regression, ‘low birth weight’ (adjusted OR (AOR) 14.7; 95% CI 2.1–101.8; *p* = 0.006), and ‘consumption of 4 or more *kuhva* per day’ (AOR 8; 95% CI 1.9–34.3; *p* = 0.005) were independently associated with infant-death-experiences.

**Conclusion:**

The present investigation merits due attention from policy makers and health planners for immediate improvement in peri-natal and neonatal care services in the remote district of Saiha. Need for further research exploring socio-behavioural issues around areca nut consumption and effects of interventions to reduce areca nut intake on maternal and children health are underscored.

## Background

Infant mortality rate (IMR) in a population specific to a year is defined as the number of deaths in children <1 year of age per 1000 live births occurring in the same year. The rate, historically regarded as a good proxy measure of population health, has been shown to bear strong association with other comprehensive measures of people’s health such as disability adjusted life expectancy [1]. Although India has witnessed a fast decline [2] in IMR in the recent past - an impressive average drop of 4.56% per year over the last 5 years - concerns have been raised on substantial differences in achievement in this regard between regions within the country and even between districts within a State [3, 4].

**Fig. 1:**
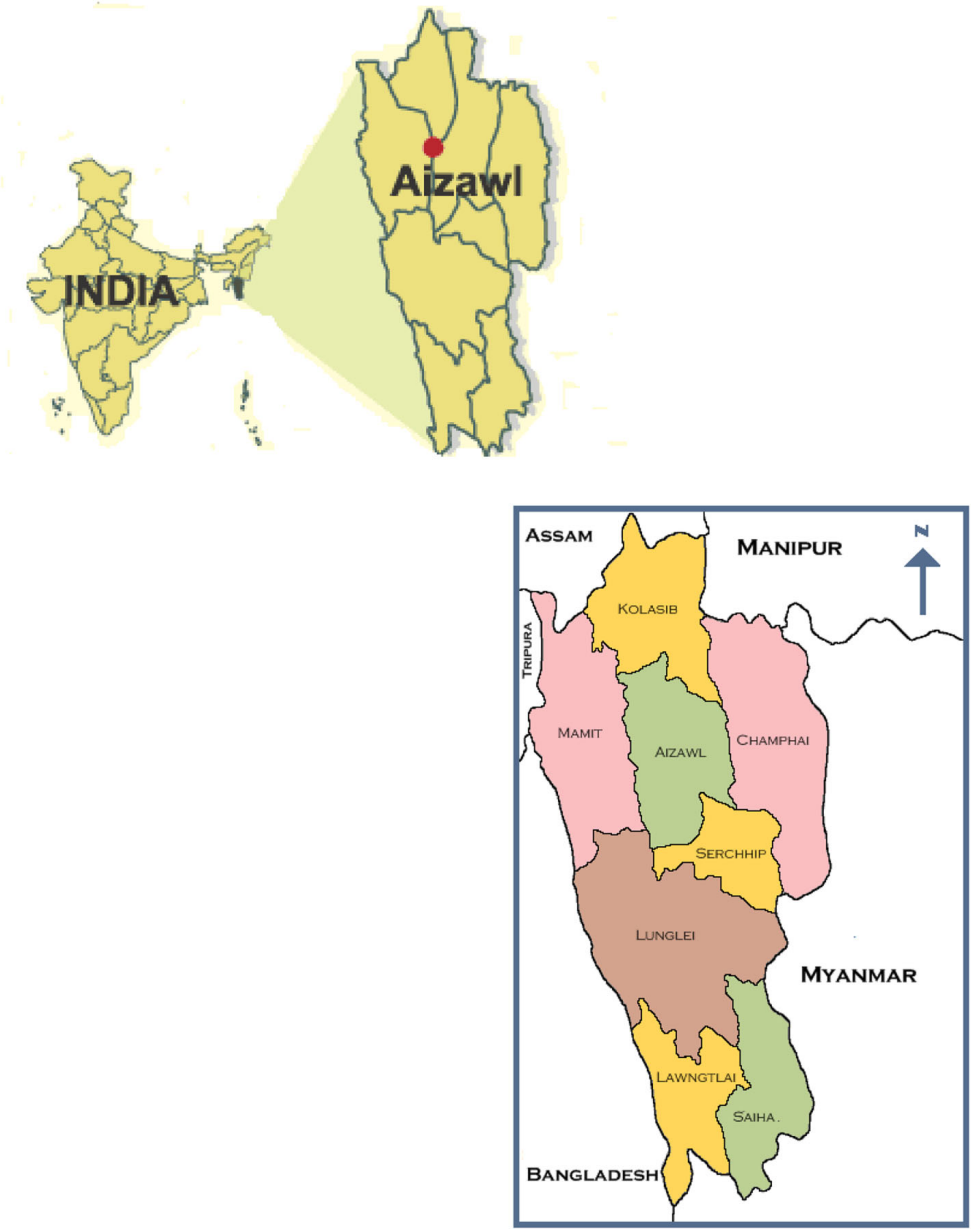
Geographical location of the study district in relation to India & neighbouring countries

Mizoram, one of the eight northeastern Indian states, shares international border with Myanmar on its east and Bangladesh on the west (Fig. 1). The crude death rate, the crude birth rate and the total fertility rate of Mizoram are below the corresponding national averages, thereby indicating a better health status in the state [5]. IMR in Mizoram varied from 30 to 37 per 1000 live births during 2012 to 2015. However disaggregated data revealed that among nine districts of Mizoram, Saiha, the southernmost district, had astonishingly high IMR of 113 per 1000 live births in the year 2014–2015 [6]. Qualitative interviews with mothers of a few deceased children by an Indian Council of Medical Research (ICMR) regional research centre during preliminary investigation in March, 2015 highlighted issues around meningitis, pneumonia, and septicemia as the probable causes of infant deaths. The report of this inquiry also mentioned about poor environmental hygiene and highlighted lack of skilled health care staff at Saiha district hospital.

We, on behalf of the National Institute of Cholera & Enteric Diseases (NICED), a premier institute of ICMR located in Kolkata, undertook a wider and in-depth community based investigation to explore issues around high infant mortality in Saiha. This was carried out at the behest of the Department of Health Research, Ministry of Health & Family Welfare and the Ministry of Tribal Affairs, Government of India. Rapid situation and response assessment (RSRA) technique [7, 8] was employed. The overall purpose of our investigation was to identify factors associated with infant mortality in this remote corner of the country and to inform the state health authority about intervention measures. NICED had ethical approval from the ‘Institutional Ethics Committee, National Institute of Cholera & Enteric Diseases’ as well as approval from the district health authority of Saiha for undertaking this research.

## Methods

### Study participants

The current investigation was conducted during 16–28th April, 2015. Secondary data were obtained from various reports and ‘mother and child information tracking system’ (MCTS registry) made available to us by the local office of the national health mission (NHM). Primary data was generated through one-on-one interviews. Mothers, who delivered a child during 2013–2015 and subsequently lost them due to infant death, formed the case group and the controls were the mothers residing in same neighborhood as with case-mothers who delivered a child during the same time frame but did not subsequently experience infant death. We planned to include two randomly selected controls against each case mother within a defined geographical neighborhood (locally termed *veng*) enlisted in the aforementioned MCTS registry. Seventy nine percent of the 86 eligible mothers in the case group (absolute number 68) and 98% of randomly selected eligible controls - both identified through MCTS - could be traced in the community for interviews. However, as appropriate locality matched controls could not be found for two cases we restricted our analysis to 66 cases. In 63 *veng-*matched case control pairs, each case was compared against two controls and in 3 pairs each case had single control.

### Study tool

An interview schedule was developed based on our reading of preliminary investigation reports generated by the regional research centre of ICMR located in one of the northeastern states of India and following consultation with the local health care providers as well as youths, adults and people having influence in the community. The interview schedule had sections on ‘socio-demography’, ‘household cooking practice’ (to explore links between solid fuel use and ailments in children such as respiratory difficulty), ‘ante-natal care related issues’, ‘health seeking practices in posed hypothetical situations about child illness’, ‘substance use during last pregnancy’ and ‘sexually transmitted disease (STD) symptoms’. Information retrieved from MCTS on ‘birth weight of children’ and ‘anemia in mothers during last pregnancy’ were incorporated in the data base.

Questionnaires used to conduct interviews were translated in local Mara dialect. It is worth noting here that Saiha district is also called Mara-land as it is predominantly inhabited by the Mara tribe of Mizoram. Informed consent was obtained from each of the mothers before interviewing them.

### Statistical analyses

Secondary data obtained from the local NHM office of Saiha, Mizoram were examined for trend in infant death (if any). Primary data captured in hard copies through interviews were checked for quality on a daily basis and computerized following necessary corrections. Unit of analysis was *veng*-matched pairs of cases and controls. Association between key risk factors and infant death experienced by mothers in the recent past (2013–2015) were examined through Mantel-Haenszel estimate of odds ratio. Biologically plausible variables with relevance for intervention development and variables bearing significant association (*p* < 0.1) with the study outcome (mothers experiencing infant-death) in this analyses were entered into a conditional logistic regression model. STATA SE version 8.2 and Epi-Info (version 6.4b) were used for data analyses.

## Results

### Secondary data on infant death

Infant death audit over the last 5 years conducted by the ‘National Health Mission’ (NHM) team in Saiha helped prepare a list of system specific ailments such as pneumonia, birth asphyxia, meningitis, diarrhea and septicemia as prime causes of deaths. Examination of ‘age at death’ revealed that about a third of the infant deaths during 2014–2015 was contributed by neonates (aged ≤ 4 weeks). This was considerably high compared to neonatal contribution of 8% to infant deaths during 2012–2013 and 15% during 2013–2014 (χ^2^ value for linear trend 41.02; *p* < 0.001). Most of the infant deaths occurred during the winter season of January through March and in ensuing fall - a trend that did not change over the years. Infant deaths occurred in almost equal proportion in males and females.

### Respondents interviewed to generate primary data

A total of 195 mothers were interviewed; 66 belonged to ‘cases’ and 129 were ‘controls’. The mean age of the respondents was 27 years (median 27; SD ± 5; minimum 17 & maximum 44 years). Of the 195 respondents, 150 were interviewed from Saiha and the rest were from the neighbouring Tuipang block - the two constituent administrative divisions of the study district.

### Household practices & socio-demographic profile

Reportedly 31 of 66 deceased infants were males (47%) and the gender distribution among live infants born to control mothers was similar (males 60/129; 47%). Case and control mothers did not differ significantly in terms of household practices (Table 1). In both the groups, a little over third reported using only the water supplied by public health engineering (PHE) department for drinking purpose. Some of the respondents reported using multiple sources of drinking water. Commonly reported combination of drinking water sources were PHE supplied water and *tuikhur* (hill stream drained in a cemented open structure and serving as public water collection point) or other sources such as water tanker, rain water, river and stream water. No difference was observed while cases and controls were compared for their usage of mixed sources for collecting drinking water. Most (~89%) of the respondents reported treating drinking water in some way or other; proportion of which did not differ significantly between case group and control.

**Table 1:** Childbirth & household characteristics of case and control groups

Factors	Categories	Cases n/N (%)^a^	Controls n/N (%)	OR^b^ (95% CI^c^ of OR)	*p* value
Place of birth during last pregnancy	Health facility	50/66 (75.8)	106/128 (82.8)	Reference	
	Home	16/66 (24.2)	22/128 (17.2)	2.2 (0.8–5.9)	0.096
Birth order at last pregnancy	1^st^	15/66 (22.7)	37/129 (28.7)	Reference	
	2^nd^–3^rd^	28/66 (42.4)	54/129 (41.9)	1.1 (0.5–2.1)	0.850
	4^th^ or more	23/68 (34.8)	38/129 (29.5)	5.3 (0.7–40.2)	0.069
Birth weight of infants born out of last pregnancy	Normal	53/62 (85.5)	84/88 (95.4)	Reference	
	Low (<2.5Kg)	9/62 (14.5)	4/88 (4.6)	2.8 (0.8–9.7)	0.095
Water fed to the infant in concern with breast milk within 6 months of life	No	56/66 (84.8)	109/129 (84.5)	Reference	
	Yes	10/66 (15.2)	20/129 (15.5)	1.0 (0.4–2.4)	0.943
Source of drinking water	Only PHE	26/66 (39.4)	46/129 (35.7)	Reference	
	Other sources	40/66 (60.6)	83/129 (64.3)	0.8 (0.4–1.7)	0.606
Domestic treatment of drinking water	Yes	57/66 (86.4)	116/129 (89.9)	Reference	
	No	9/66 (13.6)	13/129 (10.1)	1.5 (0.5–4.5)	0.469
Place for cooking	Separate kitchen	14/66 (21.2)	32/129 (24.8)	Reference	
	Next to bedroom /living room	52/66 (78.8)	97/129 (75.2)	1.3 (0.6–2.8)	0.555
Fuel used for cooking	Gas and/or Heater	21/66 (31.8)	41/129 (31.8)	Reference	
	Other fuel	45/66 (68.2)	88/129 (68.2)	1.1 (0.5–2.3)	0.894
Having pets	None	42/66 (63.6)	86/129 (66.7)	Reference	
	At least one pet	24/66 (36.4)	43/129 (33.3)	1.1 (0.6–1.9)	0.681
Having animals for sell / sacrifice at home	None	24/66 (36.4)	60/129 (46.5)	Reference	
	At least one animal	42/66 (63.6)	69/129 (53.5)	1.5 (0.8–2.7)	0.164

^a^ *%* column percentage, ^b^ *OR* odds ratio, ^c^ *CI* confidence interval

About a quarter of the households reportedly had a separate kitchen; others cooked food either adjacent to bedroom or in a corner of the living room (Table 1). Types of fuel used for cooking was distributed equally between cases and controls. Solid fuel usage, defined by wood and/or charcoal use, was reported by 65% of cases and 62% of controls.

The proportion of mothers who worked for a living was comparable among cases and controls. There was also no difference in the working status of the husbands of case and control groups (59 and 55% working, respectively). However, proportionally more mothers in case group were engaged in farming or unskilled work compared to controls (9 and 0% respectively). Greater proportion of mothers in the control group were either employed in the health or education sector or were engaged in government job compared to cases (11 and 6% respectively). Further, in higher proportion of cases, both wives and husbands reportedly were engaged in work to make a living (Table 2).

**Table 2:** Socio-demographic and pregnancy related issues in case and control groups

Factors	Categories	Cases n/N (%)	Controls n/N (%)	OR (95% CI of OR)	*p* value
Current age (years) of mother	Mean (± SD)	27.7 (5.2)	26.9 (5.2)	1.03 (0.9–1.1)	0.316
Mothers’ school Attendance	Yes	59/66 (89.4)	126/129 (97.7)	Reference	
	No	7/66 (10.6)	3/129 (2.3)	11.5 (1.1–118.2)	0.009
Grades of schooling of mothers	≥Secondary	31/66 (46.9)	80/128 (62.5)	Reference	
	<Secondary (9–10)	35/66 (53)	48/128 (37.5)	2.3 (1.1–4.9)	0.027
Mother works for a living	No	54/66 (81.8)	113/129 (87.6)	Reference	
	Yes	12/66 (18.2)	16/129 (12.4)	1.6 (0.7–3.7)	0.248
Parents’ working status	None/one working	59/66 (89.4)	125/129 (96.9)	Reference	
	Both working	7/66 (10.6)	4/129 (3.1)	4.3 (1.1–17.6)	0.025
Age at first pregnancy (years)	Mean (± SD)	21.2 (4.1)	21.3 (3.5)	0.99 (0.9–1.1)	0.904
Status as an adult during first pregnancy	Adult	55/66 (83.3)	113/129 (87.6)	Reference	
	Minor (<18y)	11/66 (16.7)	16/129 (12.4)	1.4 (0.5–3.9)	0.453
Anaemia during last pregnancy (Hb level <11 g/dl)	No	16/47 (34)	23/74 (31.1)	Reference	
	Yes	31/47 (65.9)	51/74 (68.9)	1.1 (0.4–3)	0.806
Blood transfusion during last pregnancy	No	59/66 (89.4)	127/129 (98.5)	Reference	
	Yes	7/66 (10.6)	2/129 (1.5)	6.7 (1.3–33.8)	0.007
Use of tobacco (chewing / smoking / drinking) during last pregnancy	No	9/66 (13.6)	20/129 (15.5)	Reference	
	Yes	57/66 (86.4)	109/129 (84.5)	1.2 (0.5–2.5)	0.713
*Kuhva* consumption during last pregnancy	<4 /day	15/66 (22.7)	57/129 (44.2)	Reference	
	≥4 /day	51/66 (77.3)	72/129 (55.8)	3 (1.4–6.4)	0.003

### Child birth from last pregnancy

While 70% of the children reportedly were born in the district hospital, 8% were delivered at home. Only one infant was born in a private hospital and the others were born either in local primary health centres (PHC) or in sub-centres (SC). A little over one fourth (27%) of the infants belonged to the first order of birth and a third (33%) were of birth order 4 or more. Overall, 9% of the infants had low birth weight (<2500 g). As shown in Table 1, the proportions of infants who were delivered at home or infants who had low birth weight were higher among cases compared to that in controls. Table 2 shows that the mothers in both case and control groups were similar in age at first pregnancy, although a slightly higher proportion of mothers in the case group were ‘minor’ (<18 years) while they became first pregnant. Significantly higher proportion of cases did not ever go to schools compared to controls and those among cases who attended schools had lesser standard of completed education.

### Iron and folic acid supplement during last pregnancy

Ninety four percent of mothers in both the groups (cases and controls) reportedly received iron and folic acid tablets from government health care facilities during last pregnancy. However, 76% of case-mothers and 78% of controls reportedly ingested iron tablets; the percentages were even less pertaining to intake of folic acid tablets (62 and 64% respectively). Commonly cited reasons by women for not taking these tablets were gastric irritation and nausea/vomiting. Anaemia (defined as having a haemoglobin level below 11 g/dl during pregnancy) was present in about two third of the mothers in both the groups. However, significantly more mothers in the case group reported receiving blood transfusion before or during the last delivery (surrogate of severe morbidity).

### Substance use and self-reported STD symptoms during last pregnancy

**Fig. 2:**
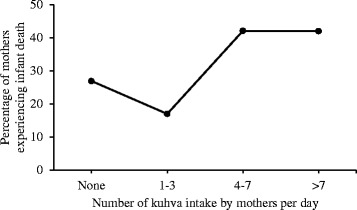
Percentage of mothers experiencing infant deaths plotted against the number of *khuva* intake per day by mothers while pregnant

Information on various substance use during last pregnancy were collected from mothers to examine if any of these practices was associated with infant loss. Except for *kuhva* (local term for raw areca nuts) intake, no significant difference was observed between case and control-mothers with regard to use of alcohol (3% vs. 0%), smoking tobacco (21% vs. 12%), chewing tobacco (79% vs. 76%), drinking *tuibur* (local nicotine-water drink) (24% vs. 26%), or use of *any* of the aforementioned tobacco products (86% vs. 84%). While median number of intake of *kuhva* per day among case-mothers during last pregnancy was 7 (IQR 4; 14), that in control mothers was 5 (IQR 2; 8) - a significant difference (OR 1.04; 95% CI 1.01–1.09; *p* = 0.016). Infant deaths plotted against per day intake of *kuhva* by mothers during last pregnancy revealed an increasing trend of deaths while mothers with no *khuva* intake served as reference (*p* = 0.006; Fig. 2). Consumption of 4 or more *kuhva* per day during last pregnancy was reported by significantly higher proportion of case-mothers (Table 2) compared to controls.

Eight percent mothers (15/195) reported experiencing genital ulcer during last pregnancy and this did not differ significantly between cases and controls. The groups of cases and controls also did not differ significantly in terms of self reported anal ulcers (1.5% vs 0%) and genital growths (0% vs 1.5%). None reported experiencing anal growth during the last pregnancy. Burning sensation during urination, although reported by a higher proportion of respondents (17/195), compared to the aforementioned genital symptoms, it did not attain any significant difference between case group and control (14% vs 6%).

### Multivariate analysis

Factors found associated with infant deaths through uni-variate analysis (*p* ≤ 0.1) and having biological plausibility were entered simultaneously in a multi-variate model. In case of two explanatory variables, which had obvious similarity, one was selected over the other. For example, we entered ‘grades of completed schooling by mothers’ in multivariate model instead of ‘never attendance to school’ although both these variables were found to be associated with infant deaths. In multivariate model, subsequent to adjustment for statistically and contextually relevant variables (shown in Table 3), the conditional logistic regression highlighted that the odds of ‘low birth weight of newborns’, and ‘consumption of 4 or more *kuhva* per day during last pregnancy’ were higher (*p* < 0.05) among mothers experiencing infant death compared to control mothers. Another factor, which, although had slightly weaker association (*p* = 0.06), also was indicated in conditional logistic regression to be associated with infant death was ‘child delivery at home’.

**Table 3:** Factors independently associated with experience of infant deaths in mothers

Factors & categories		Adjusted OR (95% CI of OR)	*p* value
Place of birth during last pregnancy	Health facility	Reference	
	Home	5.8 (0.9–36.8)	0.064
Birth order at last pregnancy	1^st^	Reference	
	2^nd^–3^rd^	2.1 (0.8–5.9)	0.151
	4^th^ or more	1.3 (0.4–4.5)	0.62
Birth weight of infants during last pregnancy	Normal	Reference	
	Low (<2.5 Kg)	14.7 (2.1–101.8)	0.006
Grades of schooling of mothers	≥Secondary	Reference	
	<Secondary (9–10)	1.8 (0.6–5.6)	0.332
Parents’ working status	None/one working	Reference	
	Both working	3.9 (0.7–22.2)	0.127
Blood transfusion during last pregnancy	No	Reference	
	Yes	3.7 (0.3–47.2)	0.312
*Kuhva* consumption during last pregnancy	<4 /day	Reference	
	≥4 /day	8 (1.9–34.3)	0.005

## Discussion

The present investigation has been able to identify factors associated with infant mortality in the southernmost district of Saiha in Mizoram, India and have implications for intervention development. We explored both distantly located as well as factors proximal to the event of infant deaths. ‘Low birth weight’ and ‘child delivery at home’ have been found in our study to be associated with experience of infant deaths in mothers. These findings are on par with the large nationally representative mortality survey examining 10892 deaths in neonates and 12260 deaths in children aged 1–59 months. Three causes accounted for 78% of all neonatal deaths in this national survey, a) prematurity and low birth weight, b) neonatal infections comprising pneumonia, sepsis and central nervous system infections and c) birth asphyxia and birth trauma [9].

We did not identify any difference between case and control groups regarding ‘exclusive breastfeeding practices’. More than 4/5th mothers in both the groups fed their children exclusively on breast during the first 6 months of life. Systematic review and critical appraisals have clearly underscored the benefits of early and exclusive breast feeding in reducing neonatal and childhood mortality resulting from various infections [10, 11].

Although mothers’ education as one of the distant and structural factors, was associated in our study, with infant death in uni-variate analysis, it was not independently associated with the outcome of interest in conditional logistic regression. Contrastingly a large population-based study in Nepal reported such association [12] and identified the protective role of maternal and paternal education against early infant death (within 24 weeks of life). Moreover, a geospatial analysis in India reported that geographic regions, which were underprivileged in child nutrition or wealth or female literacy, were also likely to be disadvantaged in terms of infant and child survival irrespective of the state to which they belonged [3]. It is important to recognize in this regard that most of the 1399 km^2^ terrain of Saiha district is hilly and travel by road from the capital city of Aizawl (about 360 km away) takes 12 h [13]. Moreover of the nine districts of Mizoram (Fig. 1), Saiha and the neighbouring district of Lawngtlai in the south rank lowest in human development index [5].

Pneumonia, meningitis, diarrhoea and septicaemia were the top four causes of infant death in Saiha as revealed through secondary data analysis. A limited follow up survey conducted over a period of 3 days in 550 children aged ≤5 years in the township of Saiha during the present investigation also revealed 23 cases of diarrhoea. A test-strip based rapid diagnostic method identified presence of rotavirus and adenovirus in about one third of these stool specimens [14]. One fourth of the stool specimens had presence of both rotavirus and adenovirus.

It was intriguing to observe a dose response relationship between ‘per day intake of raw areca nuts’ by mothers while pregnant and subsequent infant deaths. Four or more *kuhva* intake per day during pregnancy was associated with infant death in present investigation. Literature review also highlights ill effects of areca nut on health [15] and fewer benefits [16]. Cardiovascular, cerebro-vascular, hepatic and metabolic disorders including type 2 diabetes mellitus were some of the health hazards that we came across during this search. Association of oesophageal inflammation and fibrosis, respiratory discomfort, renal impairment and adverse birth outcome have also been on record [15, 17]. We underscore that the issue needs further exploration as experiments in mice have revealed toxic effects of arecoline (the choline-ester present in areca nut) on embryogenesis [18]. The World Health Organization (WHO) maintains that mild thiamine deficiency can be seen in people who have high carbohydrate and low thiamine intakes in emergency situations e.g. in individuals whose staple food is polished rice, especially if their diet contains anti-thiamine factors such as tea, coffee, betel nuts and raw fermented fish. The document also highlights the link [19] between population level thiamine deficiency and infant deaths mostly occurring between 2nd and 5th month of life. It is important to note that as per NHM record, about a third of the infant deaths in Saiha was contributed by neonates (≤4 weeks), which could therefore be linked to causes other than thiamine deficiency.

Interestingly, betel nut consumption among pregnant women has been found to be associated with folate deficiency in the neighbouring country of Bangladesh. The author of this study duly highlighted the link of such deficiency with potential adverse pregnancy outcome [20]. On the other hand a large cohort study of 4963 pregnant women on Thai-Myanmar border did not find any association between betel nut intake and adverse pregnancy outcome. The authors [21] however specified that the Karen and Burmese pregnant women (their study population) used lower number of ripe nuts per day - a practice very different from Papuans and Taiwanese (covered in earlier studies producing contradictory result) who reportedly used higher number of unripe nuts [22, 23].

### Limitations

The present investigation had suffered from some limitations. First of all, the nature of investigation necessitated asking mothers if they lost their child within infancy who was born out of last pregnancy (confirming the ‘case’ and ‘control’ status to avoid false classification). This did not allow ‘masking’ the interviewers about case-control status of the participants. Secondly causes of deaths in infants were not ascertained with certainty in the remote district of Saiha precluding in-depth analysis of the same. Lastly due to lack of meticulous health record keeping at the level of individual participants, we had to take resort to using MCTS-data base maintained by NHM. Despite such limitations, the current study has highlighted a few intervention areas of public health importance. Participation of local stakeholders including state health authority in the investigation was crucial in achieving such feat.

## Conclusions

We have identified two proximate attributes of infant deaths namely ‘low birth weight’, and ‘raw areca nut (*kuhva*) intake’ in the southernmost district of Saiha, in Mizoram - a northeastern state of India bordering Myanmar. Child delivery at home has also been indicated in our study as one of the elements that should be brought under the folds of intervention. While these findings are similar to investigations conducted in other parts of the country and in similar settings of south and southeast Asia, association of a dose response relationship between unripe areca nut intake during pregnancy and infant death is intriguing. The present investigation merits due attention from policy makers and health planners for immediate improvement in peri-natal and neonatal care services. The need for further research, especially exploring socio-behavioural issues around areca nut consumption and effects of intervention to reduce areca nut intake on the health of women, children and men are underscored.

## Abbreviations

AOR: Adjusted odds ratio
CI: Confidence interval
ICMR: Indian Council of Medical Research
IMR: Infant mortality rate
MCTS: Mother & child tracking system
NHM: National Health Mission
NICED: National Institute of Cholera & Enteric Diseases
OR: Odds ratio
PHC: Primary Health Centre
PHE: Public Health Engineering
RSRA: Rapid situation & response assessment
SC: Sub-centre
SD: Standard deviation
STD: Sexually transmitted diseases
VIP: Very important person
WHO: World Health Organization

## References

[1] Reidpath DD, Allotey P (2003). Infant mortality rate as an indicator of population health. J Epidemiol Community Health.

[2] Planning Commission. Estimates of birth rate, death rate, natural growth rate, infant mortality rate and total fertility rate by residence, 1972-2013. Data-book for use of deputy Chairman, Planning Commission, Government of India; 2014. p. 182.

[3] Singh A, Pathak PK, Chauhan RK, Pan W (2011). Infant and child mortality in India in the last two decades: a geospatial analysis. PLoS One.

[4] Patel TA, Sharma DB (2013). Interstate variation in neonatal mortality rate among Indian states. National J Comm Med.

[5] Institute for Human Development & Government of Mizoram. Mizoram Human Development Report, 2013. New Delhi: Institute for Human Development; 2014.

[6] IMR & MMR, Mizoram: District wise status of maternal death & infant death, 2015. Health & Family Welfare Department, Government of Mizoram. https://health.mizoram.gov.in/page/imr-mmr Accessed 23 May 2015.

[7] Chambers R (1981). Rapid rural appraisal: rationale and repertoire. Public Admin Develop.

[8] Manderson L, Aaby P (1992). An epidemic in the field? Rapid assessment procedures in health research. Soc Sci Med.

[9] The Million Death Study Collaborators (2010). Causes of neonatal and child mortality in India: a nationally representative mortality survey. Lancet.

[10] Debes AK, Kohli A, Walker N, Edmond K, Mullany LC (2013). Time to initiation of breast feeding and neonatal mortality and morbidity: a systematic review. BMC Public Health.

[11] Dadhich JP, Agarwal RK (2009). Mainstreaming early and exclusive breastfeeding for improving child survival. Indian Pediatr.

[12] Katz J, West KP, Khatry SK, Christian P, LeClerq SC, Pradhan EK (2003). Risk factors for early infant mortality in Sarlahi district, Nepal. Bull World Health Organ.

[13] Sundaram A (2009). A study of socio-economic analysis of tribal households in Saiha district of Mizoram.

[14] National Institute of Cholera & Enteric Diseases (2015). Health investigation report : Infant mortality in the district of Saiha, Mizoram. Report for the Indian Council of Medical Research.

[15] Javed F, Correa FOB, Chotai M, Tappuni AR, Almas K (2010). Systemic conditions associated with areca nut usage: a literature review. Scand J Public Health.

[16] Lingappa A, Nappalli D, Sujatha GP, Shiva Prasad S (2011). Areca nut: to chew or not to chew. EJ Dent.

[17] Garg A, Chaturvedi P, Gupta PC (2014). A review of the systemic adverse effects of areca nut or betel nut. Indian J Med Ped Oncol.

[18] Liu S-T, Young G-C, Lee Y-C, Chang Y-F (2011). A preliminary report on the toxicity of arecoline on early pregnancy in mice. Food Chemical Toxicol.

[19] World Health Organization (1999). Thiamine deficiency and its prevention and control in major emergencies WHO/NHD/99.13 (English/General).

[20] Kader M (2013). Association between betel nut consumption and folate deficiency among pregnant women in rural Bangladesh. Int J Med Public Health.

[21] Chue AL, Carrara VI, Paw MK, Pimanpanarak M, Wiladphaingern J, Vugt MV (2012). Is areca innocent? The effect of areca (betel) nut chewing in a population of pregnant woman on the Thai-Myanmar border. Int Health.

[22] Taufa T (1988). Betel-nut chewing and pregnancy. PNG Med J.

[23] Yang MJ, Chum TC, Yang MJ, Hsu TY, Ko YC (2001). Betel-quid chewing and risk of adverse birth outcomes among aborigines in eastern Taiwan. J Toxicol Environ Health A.

